# Complications of stent placement in patients with esophageal cancer: A systematic review and network meta-analysis

**DOI:** 10.1371/journal.pone.0184784

**Published:** 2017-10-02

**Authors:** Amin Doosti-Irani, Mohammad Ali Mansournia, Abbas Rahimi-Foroushani, Peiman Haddad, Kourosh Holakouie-Naieni

**Affiliations:** 1 Department of epidemiology, school of public health, Hamadan University of medical sciences, Hamadan, Iran; 2 Department of epidemiology and biostatistics, school of public health, Tehran University of medical sciences, Tehran, Iran; 3 Radiation Oncology Research Center, Cancer Institute, Tehran University of Medical Sciences, Tehran, Iran; University Hospital Llandough, UNITED KINGDOM

## Abstract

**Background:**

Palliative treatments and stents are necessary for relieving dysphagia in patients with esophageal cancer. The aim of this study was to simultaneously compare available treatments in terms of complications.

**Methods:**

Web of Science, Medline, Scopus, Cochrane Library and Embase were searched. Statistical heterogeneity was assessed using the Chi^2^ test and was quantified by I^2^. The results of this study were summarized in terms of Risk Ratio (RR). The random effects model was used to report the results. The rank probability for each treatment was calculated using the p-score.

**Results:**

Out of 17855 references, 24 RCTs reported complications including treatment related death (TRD), bleeding, stent migration, aspiration, severe pain and fistula formation. In the ranking of treatments, thermal ablative therapy (p-score = 0.82), covered Evolution® stent (p-score = 0.70), brachytherapy (p-score = 0.72) and antireflux stent (p-score = 0.74) were better treatments in the network of TRD. Thermal ablative therapy (p-score = 0.86), the conventional stent (p-score = 0.62), covered Evolution® stent (p-score = 0.96) and brachytherapy (p-score = 0.82) were better treatments in the network of bleeding complications. Covered Evolution® (p-score = 0.78), uncovered (p-score = 0.88) and irradiation stents (p-score = 0.65) were better treatments in network of stent migration complications. In the network of severe pain, Conventional self-expandable nitinol alloy covered stent (p-score = 0.73), polyflex (p-score = 0.79), latex prosthesis (p-score = 0.96) and brachytherapy (p-score = 0.65) were better treatments.

**Conclusion:**

According to our results, thermal ablative therapy, covered Evolution® stents, brachytherapy, and antireflux stents are associated with a lower risk of TRD. Moreover, thermal ablative therapy, conventional, covered Evolution® and brachytherapy had lower risks of bleeding. Overall, fewer complications were associated with covered Evolution® stent and brachytherapy.

## Introduction

Esophageal cancer includes squamous cell carcinoma and adenocarcinoma. It has an aggressive nature, a poor prognosis and a low five-year survival rate [1]. More than 50% of patients with esophageal cancer are diagnosed with an advanced stage of the disease, for which surgery is not appropriate. Therefore, palliative treatments are necessary for relieving the associated dysphagia [2]. Certain treatment options have been developed to relieve the pain and dysphagia, including the stent, brachytherapy, and external radiotherapy [3]. Different types of stents such as Self-expandable metallic stents (SEMS), Self-expandable plastic stents (SEPS), polyflex, and antireflux, have been developed and compared in randomized control trials (RCT) [4]. However, the optimal treatment intervention has not been determined yet [3]. Up to now, many RCTs have been conducted to assess the effectiveness of stents and palliative treatments among patients with advanced esophageal cancer [5–10]. These RCTs have reported adverse events for stent placement and other palliative treatments such as brachytherapy and thermal ablative treatment in patients, including TRD, bleeding, stent migration, fistula formation, and aspiration. However, there is no consensus as to which stent has lower complications [3].

A network meta-analysis simultaneously comparing all available treatments can prove useful in the selection of better treatments [11]. Therefore, the aim of this network meta-analysis was to simultaneously compare available palliative treatments in terms of complications, including TRD, bleeding, stent migration, aspiration, severe pain and fistula formation, and to rank these treatment interventions in patients with esophageal cancer.

## Methods

### Search strategy and selection criteria

This network meta-analysis is a part of a comprehensive systematic review which has simultaneously compared all the treatment interventions for esophageal cancer. The protocol of this systematic review has been registered in PROSPERO (ID: CRD42015023950) and has also been published [12].

A search strategy was developed to obtain relevant RCTs on the evaluation of treatment interventions for esophageal cancer. The search strategy for this review included the following Keywords: #1: Esophageal cancer [tw]; #2: Esophageal squamous cell carcinoma [Mesh terms]; #3: Esophageal Neoplasms [Mesh terms]; #4: #1 OR #2 OR #3; #5: Randomized controlled trial [Mesh terms]; #6: Randomized clinical trial [tw]; #7: #5 OR #6; #8: Radiotherapy [Mesh terms]; #9: Stents [Mesh terms]; #10: Brachytherapy [Mesh terms]; #11: Palliative Care [Mesh terms]; #12: #8 OR #9 OR #10 OR #11; #13: #4 AND #7 AND #12.

The international databases searched until July 2017 included Web of Science, Medline, Scopus, Cochrane Library and Embase. During the hand-search, the reference lists of the included RCTs and relevant published systematic reviews and meta-analyses were also scanned. The authors of the included studies were contacted. In addition, the following websites of relevant conferences were searched to obtain unpublished articles:

International Gastric Cancer Association; available from http://www.igca.info/news/dec2012_02.html

The International Society for Diseases of the Esophagus; available from http://www.isde.net/events

Cancer Research UK; available from: http://www.cancercentre.ox.ac.uk/events/sponsored-events/symposium-on-oesophageal-cancer/

World Organization for Specialized Studies on Diseases of the Esophagus; available from: http://www.oeso.org/index.html

Gastroenterology Conference Map; available from: http://www.mdlinx.com/gastroenterology/conference-map.cfm

All RCTs that had evaluated stent placement or palliative treatments of esophageal cancer were retrieved in this review. We put no restriction on the time, location and language of the published RCTs. The inclusion criteria for this review were, RCTs that included patients with either histology of esophageal cancer i.e. squamous cell carcinoma and/or adenocarcinoma. Cohort studies and non-randomized clinical trials were excluded.

### Data extraction and quality assessment

Two investigators (ADI & MAM) screened the titles and abstracts of the retrieved RCTs independently. They reviewed the full texts of relevant RCTs to assess the eligibility for inclusion in the network meta-analysis. Any disagreement between the authors was resolved by discussion and -if need be- by the judgment of other authors. The following data were extracted using a predefined data sheet: year of publication, location, duration of study (months), stage of cancer, type of palliative treatment in each arm of the RCT in detail, number of randomized patients in each arm of the RCT, numbers of males and females, mean/median age of participants and the numbers of complications among patients in each arm of the included RCT. The risk of bias was assessed using Cochrane’s tools [13]. The details of used items from Cochrane’ tools in this review were reported in the protocol [12].

### Outcome

The outcomes of interest in this study were TRD, bleeding, stent migration, aspiration, severe pain and fistula formation among patients with esophageal cancer who had received palliative treatment interventions.

### Statistical analysis

In the first stage, the network of treatment interventions was drawn using the *netgraph* command in R software. We performed a pairwise meta-analysis for each comparison using the random effect model. A Risk Ratio with 95% CI was calculated for each complication in two by two comparisons. The statistical heterogeneity was assessed using the Chi^2^ test and the heterogeneity across each comparison was quantified using I^2^ statistics [14]. The similarity assumption was assessed clinically and epidemiologically in terms of effect modifiers in the arms of the RCTs. The consistency assumption was assessed using loop-specific and design by treatment interaction methods [15]. The publication bias for each complication was assessed visually by the adjusted network funnel plot using Stata 13 (Stata Corp, College Station, TX, USA) [16]. The results of this random effect network meta-analysis were summarized in terms of Risk Ratio with 95% Confidence Interval (CI).

The rank probabilities of the treatments were calculated using the p-score. The p-score for treatment *i* is one minus the one-sided p-value p[*j*] of accepting the alternative hypothesis. Therefore, the p-score for each treatment is the mean of all 1-p[j]. The P-score is a value between zero and one, where a larger value indicates a better treatment [17]. The statistical analysis was performed using R, version 3.3.1 (2016-06-21), with the netmeta package for network meta-analysis.

## Results

**Fig 1:**
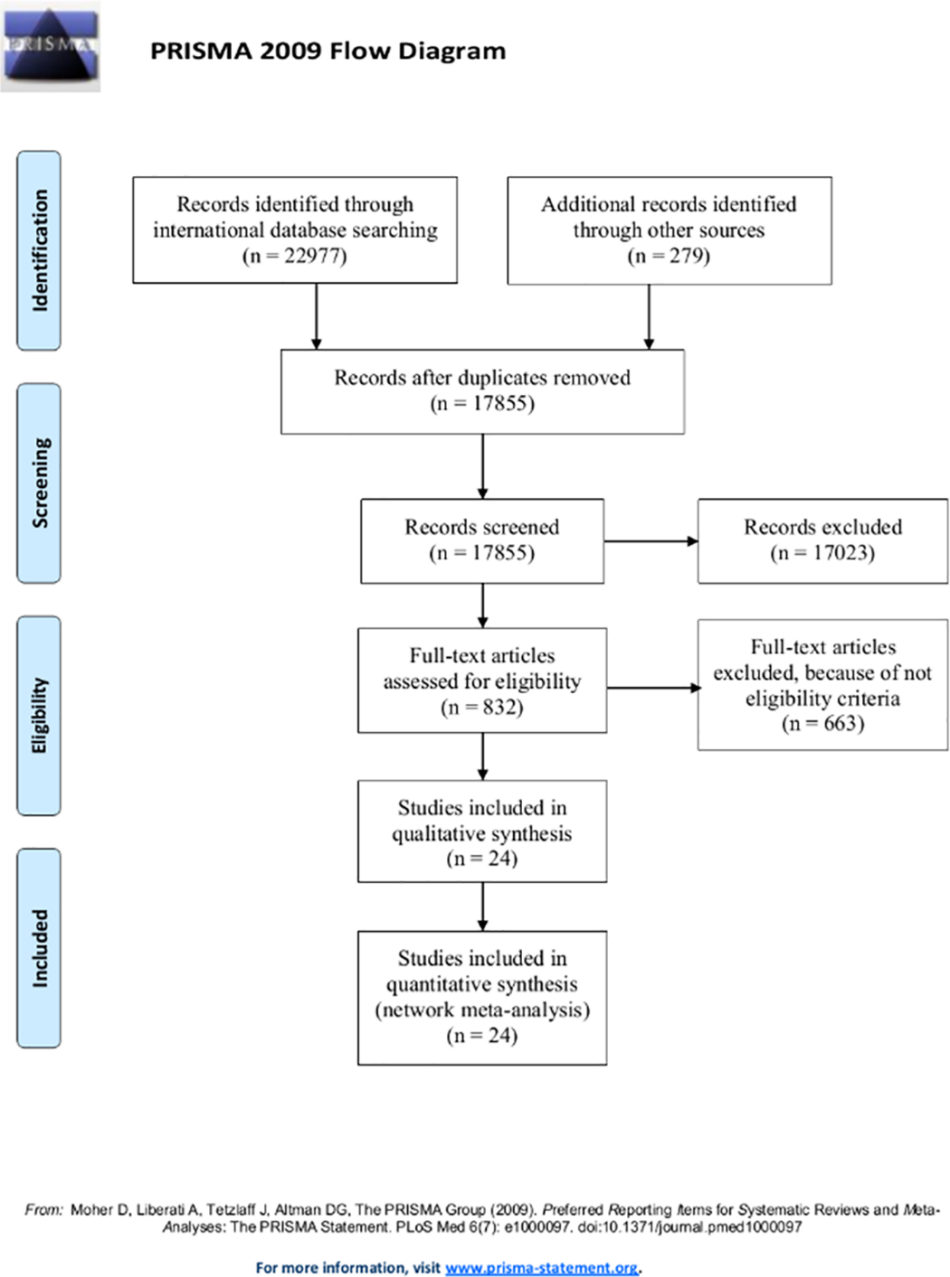
A flow chart depicting the stages of retrieving articles and checking the eligibility criteria for network meta-analysis.

Overall, 17855 references were retrieved after removing duplicate references. After screening the titles and abstracts, 832 RCTs’ full texts remained for review. Upon checking the eligibility criteria, 24 RCTs [5, 7–10, 18–36] had reported the complications of stents and palliative treatments, so they were included in our network meta-analysis (Fig 1 and Table 1).

**Table 1:** *Characteristics* of included randomized control trials.

Author	Quality	Country	Treatments	n1	n2	proportion of male in arm 1	Proportion of male in arm 2	Mean age 1	Mean age 2	TRD 1	TRD 2	Bleeding 1	Bleeding 2	Stent migration 1	Stent migration 2	Aspiration 1	Aspiration 2	Sever pain 1	Sever pain 2	Fistula 1	Fistula 2
Amdal (2013)	I	Norway	T1: SEMS+BT T2: BT	21	20	0.62	0.65	74	73			1	0	1	0	3	0				
Conio (2007)	I	International	T1: Polyflex T2: Ultraflex stent	47	54	0.83	0.83	74.9	69.1	2	4	2	0	7	1					1	2
Dallal (2001)	I	UK	T1: Thermal ablative therapy T2: Metallic stent	34	31	0.53	0.52	75	77	1	2	0	3	0	1					3	0
De Palma (1996)	L	Italy	T1: Plastic stent T2: Metallic stent	20	19	0.75	0.84	69.4	67.8	3	0	1	0								
Guo (2008)	H	China	T1: Irradiation stent T2: Conventional stent	27	26	0.70	0.77	72.2	69.5	6	5	9	7	2	3	1	2	8	7	1	0
Homs (2004)	I	Netherlands	T1: BT T2: SEMS 18	101	108	0.75	0.80	69	69	1	6	5	14	3*	18	1	1	1	3	3	3
Homs (2004)	I	Netherlands	T1: FerX-Ella+ antireflux T2: FerX-Ella	15	15	0.80	0.80	68	69	0	0	2	1	5	2	0	1	1	1		
Javed (2012)	H	India	T1: Ultraflex stent T2: Ultraflex stent+RT	37	42	0.73	0.69	58.1	58.6	0	0			9	6						
Knyrim (1993)	L	Germany	T1: Plastic stent T2: Metallic stent	21	21	0.00	0.00	68.8	64.8	6	3			5	0	1	0			1	2
O’Donnell (2002)	L	UK	T1: Plastic stent T2: Metallic stent	25	25	0.00	0.00	72.3	72.9	5	6			3	2						
Power (2007)	H	Ireland	T1: Conventional stent T2: Antireflux stent	25	24	0.68	0.58	73.9	64.8			0	0	0	2			1	2		
Sabharwal (2008)	I	UK	T1: Antireflux stent T2: Ultraflex stent + omeprazole	22	26	0.68	0.81	71.4	66.3	0	1	1	2	7	6			2	9	0	1
Sabharwal (2003)	I	UK	T1: Ultraflex stent T2: Flamingo stent	31	22	0.81	0.68	71.6	61.2	5	4	1	1	2	1						
Shenfine (2009)	H	UK	T1: Rigid plastic stent T2: Non-Stent	57	50	0.70	0.78	78.2	76.9			12	6	17	8	2	0	8	7		
Vakil (2001)	I	Italy	T1: Metallic stent T2: Uncovered stent	32	30	0.00	0.00	74	71			8	2	4	2			12	15		
van Heel (2012)	L	Netherlands	T1: Ultraflex stent T2: Covered Evolution stent	40	40	0.80	0.65	67	67	10	7	7	0	3	1	3	2	5	6		
Wenger (2006)	I	Sweden	T1: Antireflux T2: Conventional	19	22	0.68	0.59	75	73	0	0	1	1	2	3						
Wenger (2005)	H	Sweden	T1: SEMS T2: BT	30	30	0.60	0.70	74	72	2	2	1	0	2	-					2	1
White (2015)	H	Kenia	T1: SEMS 18 T2: SEMS 23	50	50	0.62	0.58	61.8	57.1					3	0			1	0		
Zhu (2014)	H	China	T1: Irradiation Stent T2: CSENACS	80	80	0.76	0.66	71	71			5	5	0	0	11	14	17	15	6	5
Laasch (2002)	L	UK	T1: Open Stent T2: Antireflex stent	25	25	0.76	0.60	69	69	1	0			3	4			1	0		
Siersema (1998)	I	Netherlands	T1: Latex prosthesis T2: Metallic stent stent	38	37	0.76	0.70	65.2	67.6	4	1	5	3	3	0			6	12	1	0
Siersema (2001)	L	Netherlands	T1: Ultraflex stent T2: Flamingo stent	34	33	0.82	0.67	67.7	71.4	1	0	5	3	6	3			3	6		
Verschuur (2008)	I	Italy	T1: Ultraflex stent T2: Polyflex stent	42	41	0.67	0.68	69	70	1	2	5	5	7	12	1	0	1	0	2	0

H: high quality; I: intermediate quality; L: low quality; T1: treatment 1, T2: treatment 2; BT: Brachytherapy; n1: sample size in arm 1, n2: sample size in arm 2; CSENACS: Conventional self-expandable nitinol alloy covered stent; SEMS: Self-expandable metallic stents.

*In this RCT some of randomized patients in BT group received SEMS 18.

**Fig 2:**
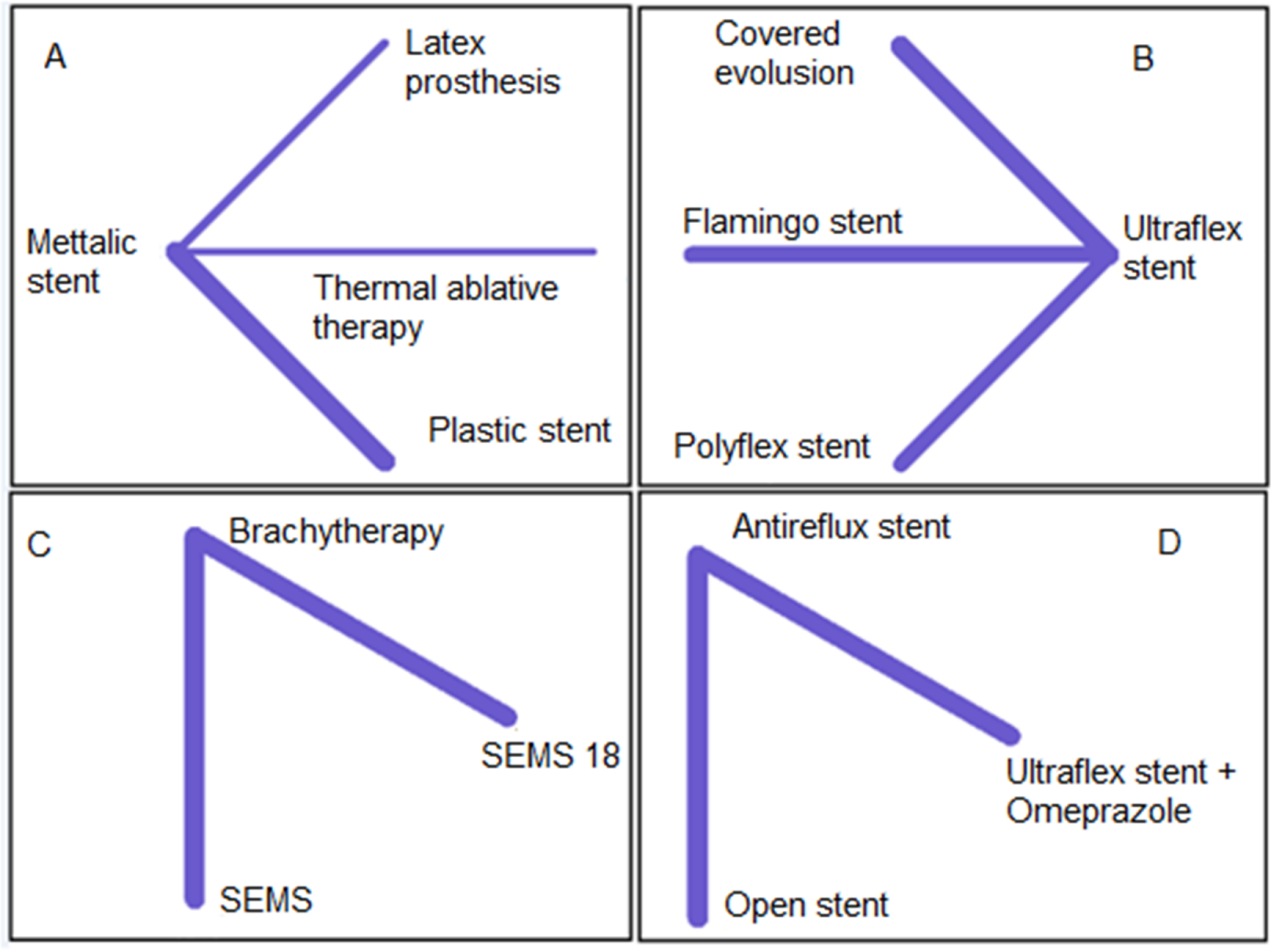
Network of stent interventions for palliative treatments that had reported TRD in esophageal cancer; A: the metallic stent is a reference treatment, B: Ultraflex is a reference, C: Brachytherapy is a reference, and D: Antireflux is a reference.

**Fig 3:**
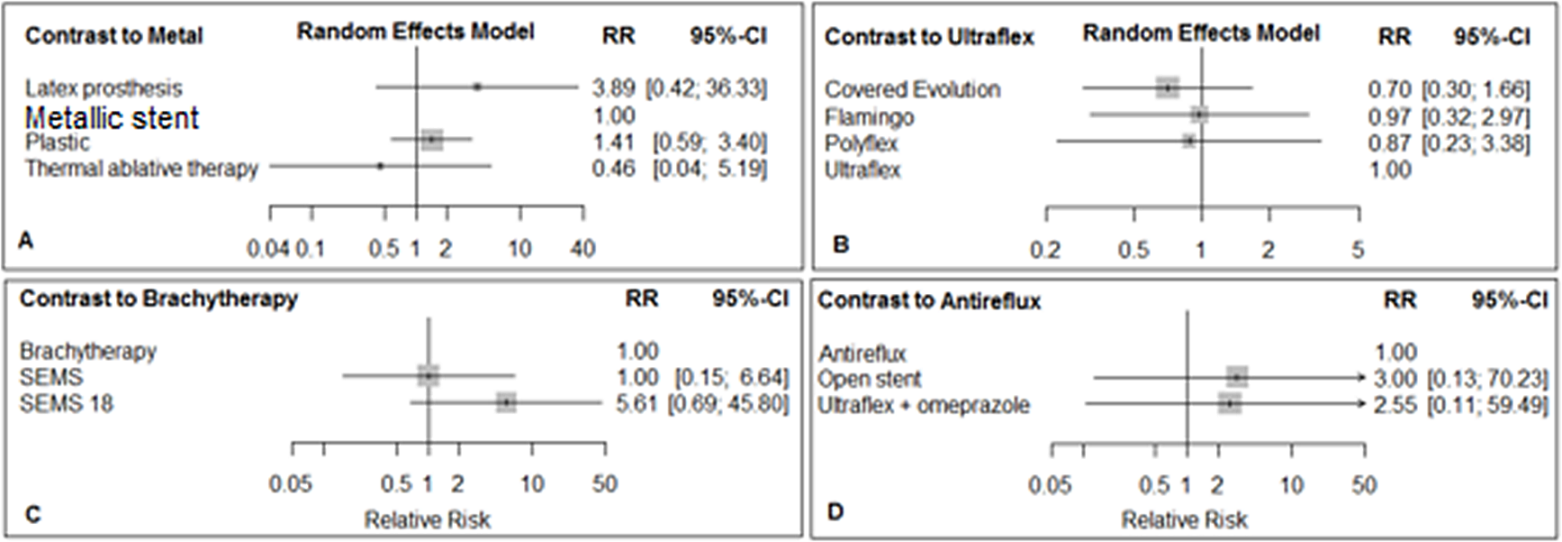
Forest plots for TRD in networks A, B, C, & D. Metallic stent, Ultraflex, Brachytherapy, and Antireflux are reference treatments in networks A, B, C & D, respectively.

As shown in Figs 2 and 3, and S1, S3, S5 and S7 Figs, there were no closed loops in the networks, so we could not assess the consistency assumption of the complications. However, we assessed the heterogeneity in the two by two comparisons and the entire networks. The results showed no significant heterogeneity in the networks in either of the complications (S1–S6 Tables). Based on the adjusted funnel plot there was no evidence of publication bias for the set of studies related to each complication (S9–S14 Figs).

### Treatment-related death complication

TRD was reported in 16 RCTs, which included 1075 patients with esophageal cancer. The networks of eligible comparisons for TRD and bleeding are shown in Figs 2 and 3. The comparisons of treatments for TRD involved four independent sub-networks. In the networks, A and B, C and D, the metallic stent, the Ultraflex stent, brachytherapy and antireflux stents were reference treatments, respectively (Fig 2). According to the results of the test for heterogeneity, the I^2^ statistic for network A was 15.2%, and for network B, C, and D was zero (S1 Table).

The comparison of palliative treatments with reference treatments in each network for TRD is shown in Fig 3. In network A, compared with the metallic stent, the latex prosthesis increased the risk of TRD. The relative risk (RR) was 3.89 (95% CI: 0.42, 36.33). The RR for thermal ablative therapy compared with the metallic stent was 0.46 (95% CI: 0.04, 5.19). In network B, covered Evolution® compared with Ultraflex stent decreased the risk of TRD, RR = 0.70 (95% CI: 0.30, 1.66). In network C, SEMS 18 compared to brachytherapy increased the risk of TRD, RR = 5.61, (95% CI: 0.69, 45.80). In network D, both the open stent and ‘Ultraflex plus omeprazole’ compared to antireflux stent increased the risk of TRD (Fig 3).

Results of simultaneous direct and indirect comparisons of treatments for TRD are shown in S1 Table.

In terms of ranking of treatments, thermal ablative therapy (p-score = 0.82), covered Evolution® (p-score = 0.70), brachytherapy (p-score = 0.72) and antireflux stent (p-score = 0.74) were the best treatments in networks A, B, C and D, respectively (Table 2).

**Table 2:** Ranking of palliative treatments in terms of lower risk of TRD and bleeding, stent migration, aspiration, severe pain and fistula in patients with esophageal cancer.

TRD							
Network A		Network B		Network C		Network D	
Treatment	P-score	Treatment	P-score	Treatment	P-score	Treatment	P-score
Thermal ablative therapy	0.82	Covered evolution	0.70	Brachytherapy	0.72	Antireflux	0.74
Metallic stent	0.65	Polyflex	0.49	SEMS	0.69	Ultraflex +omeprazole	0.40
Plastic stent	0.38	Flamingo	0.44	SEMS18	0.09	Open stent	0.36
Latex prosthesis	0.15	Ultraflex	0.37				
Bleeding							
Network A		Network B		Network C		Network D	
Treatment	P-score	Treatment	P-score	Treatment	P-score	Treatment	P-score
Thermal ablative therapy	0.86	Conventional	0.62	Covered evolution	0.96	Brachytherapy	0.82
Uncovered stent	0.79	Antireflux	0.54	Flamingo	0.50	SEMSBT	0.41
Metallic stent	0.41	CSENACS	0.49	Ultraflex	0.34	SEMS	0.40
Latex	0.24	Irradiation stent	0.47	Polyflex	0.20	SEMS18	0.36
Plastic stent	0.20	Ultraflex + Omeprazole	0.37	-	-	-	-
Stent migration							
Network A		Network B		Network C			
Treatment	P-score	Treatment	P-score	Treatment	P-score		
Covered Evolution stent	0.78	Uncovered stent	0.88	Irradiation stent	0.65		
Flamingo stent	0.67	Metallic stent	0.69	Ultraflex stent+ omeprazole	0.52		
Ultraflex stent + RT	0.66	Plastic stent	0.27	Open stent	0.50		
Ultraflex stent	0.36	Latex prosthesis stent	0.16	Conventional stent	0.49		
Polyflex stent	0.03			Antireflux stent	0.34		
Aspiration							
Network A		Network B		Network C			
Treatment	P-score	Treatment	P-score	Treatment	P-score		
Polyflex stent	0.69	Irradiation stent	0.74	BT	0.69		
Covered evolution	0.52	CSENACS	0.46	SEMS18	0.68		
Ultraflex stent	0.29	Conventional stent	0.31	SEMSBT	0.13		
Severe pain							
Network A		Network B		Network C		Network D	
Treatment	P-score	Treatment	P-score	Treatment	P-score	Treatment	P-score
CSENACS	0.73	Polyflex stent	0.79	Latex prosthesis	0.96	BT	0.65
Conventional stent	0.71	Ultraflex	0.58	Metallic stent	0.44	SEMS23	0.63
Irradiation stent	0.63	Covered evolution	0.45	Uncovered stent	0.10	SEMS18	0.22
Antireflux	0.52	Flamingo	0.19	-	-	-	-
Open stent	0.27	-	-	-	-	-	-
Ultraflex + Omeprazole	0.14	-	-	-	-	-	-
Fistula							
Network A		Network B		Network C			
Treatment	p-score	Treatment	p-score	Treatment	p-score		
Plastic stent	0.81	Conventional stent	0.72	SEMS18	0.62		
Metallic stent	0.64	CSENACS	0.46	BT	0.59		
Latex	0.36	Irradiation	0.32	SEMS	0.29		
Thermal ablative therapy	0.19	-	-	-	-		

### Bleeding

Bleeding was reported in 18 RCTs, which included 1374 patients with esophageal cancer. Other characteristics of the included RCTs are shown in Table 1.

**Fig 4:**
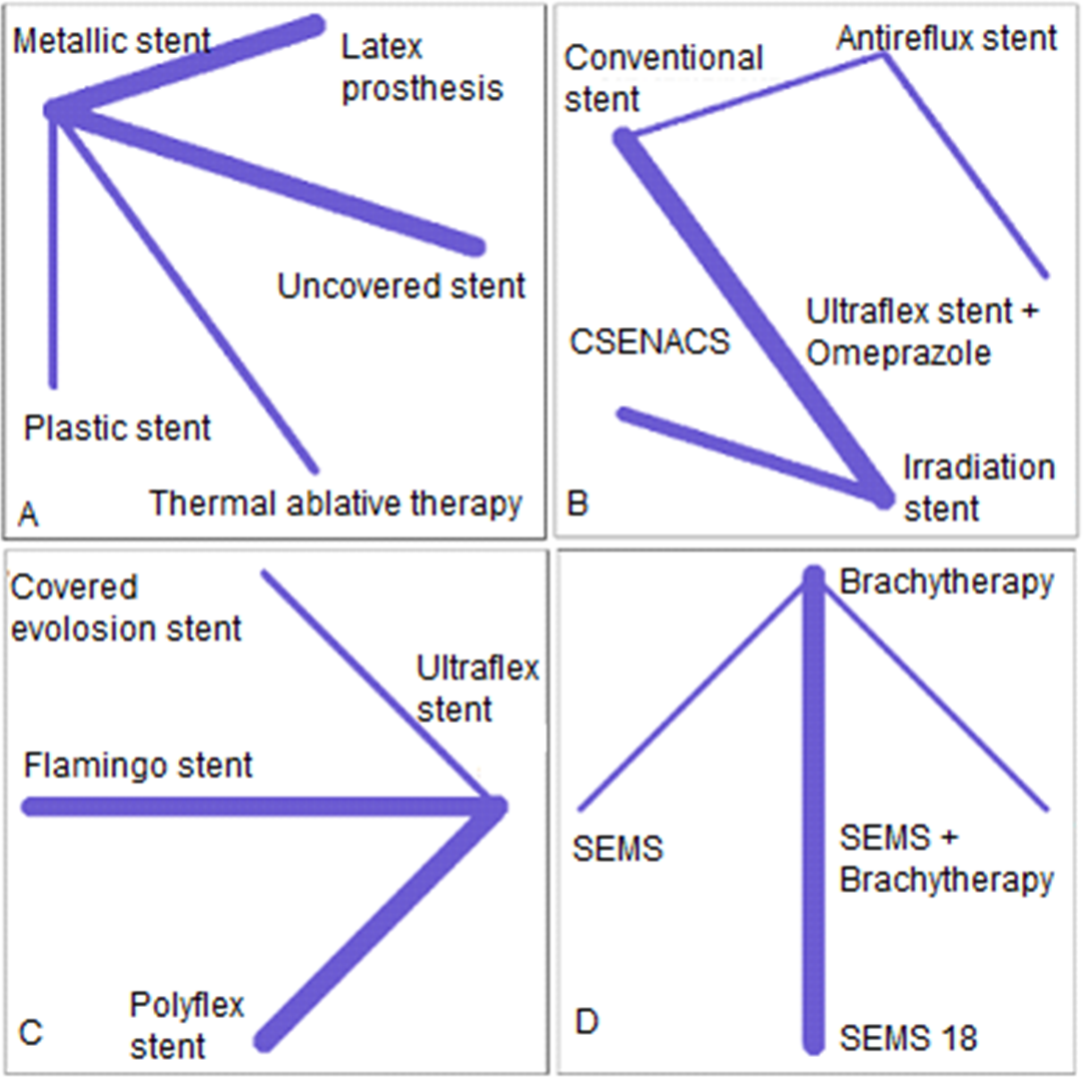
The network of stent interventions for palliative treatments that reported the bleeding complication in esophageal cancer; A: metallic stent is a reference treatment, B: Conventional is a reference, C: Ultraflex is a reference, and D: Brachytherapy is a reference treatment. CSENACS: Conventional self-expandable nitinol alloy covered stent.

In the network of bleeding complications, the metallic stent, conventional stent, Ultraflex stent, and brachytherapy were reference treatments in networks A, B, C, and D, respectively (Fig 4). Based on the results of the test for heterogeneity, the I^2^ statistic for network A, B, C, and D was zero (S2 Table).

**Fig 5:**
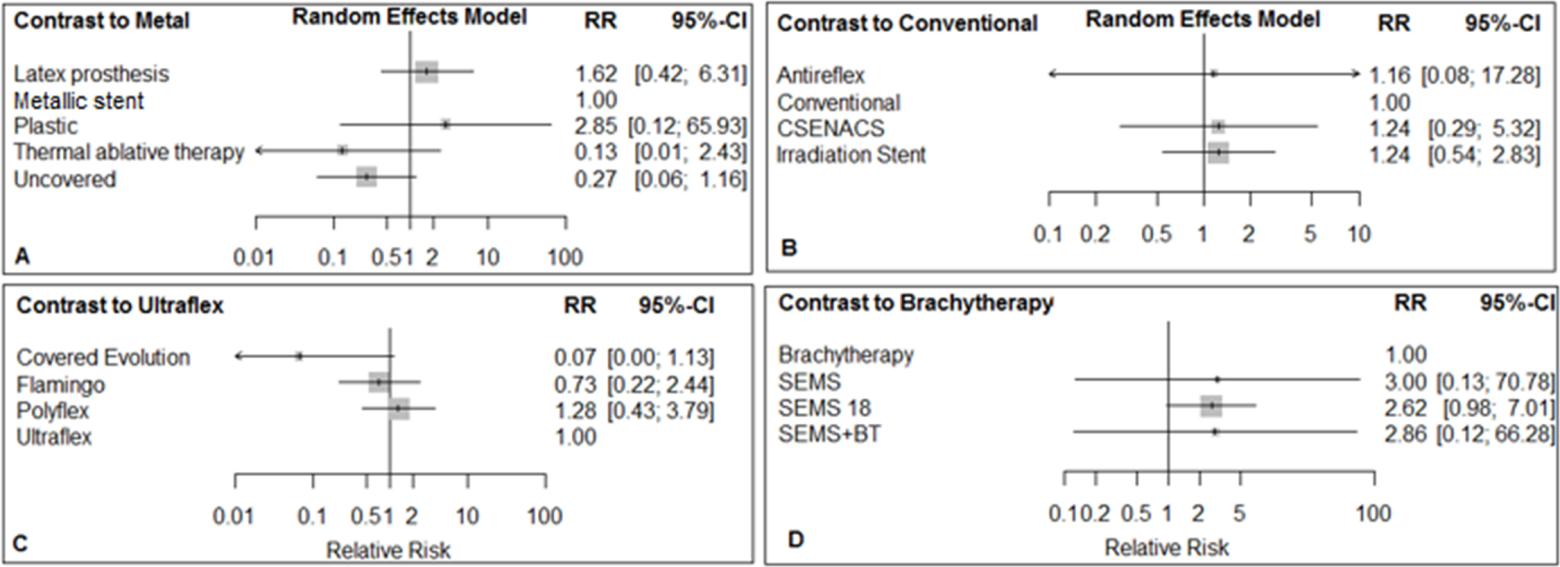
Forest plots for bleeding complication in networks A, B, C & D. Metallic stent, Conventional stent, Ultraflex, and Brachytherapy are reference treatments in networks A, B, C & D, respectively.

In network A, the latex prosthesis and plastic stent increased the risk of bleeding when compared to the metallic stent. The RR for latex prosthesis was 1.62 (95% CI: 0.42, 6.31) and was 2.85 (95% CI: 0.12, 65.93) for the plastic stent. On the other hand, thermal ablative therapy (RR = 0.13, 95% CI: 0.01, 2.43) and uncovered stent (RR = 0.27, 95% CI: 0.06, 1.16) decreased the risk of bleeding when compared to the metallic stent. In network B, the irradiation stent and CSENACS increased the risk of bleeding when compared to the conventional stent. In network C, the covered Evolution® stent decreased the risk of bleeding (RR = 0.07, 95% CI: 0.00, 1.13) when compared to Ultraflex. In network D, SEMS and SEMS+BT increased the risk of bleeding when compared to brachytherapy (Fig 5).

The results of simultaneous direct and indirect comparisons of treatments for bleeding are shown in S2 Table.

In terms of ranking treatments for the lower risk of bleeding, thermal ablative therapy (p-score = 0.86), the conventional stent (p-score = 0.62), covered Evolution® (p-score = 0.96) and brachytherapy (p-score = 0.82) were the best treatments in networks A, B, C, and D, respectively (Table 2).

### Stent migration

Stent migration was reported in 23 RCTs. However, in 4 of the RCTs patients had received BT or thermal ablative therapy [5, 8, 19, 34] in one arm. Since stent migration cannot be a complication of BT and thermal ablative therapy, these RCTs were excluded from the network meta-analysis. Therefore, 19 RCTs involving 1207 patients were included. The available treatments for stent migration involved three dependent networks (S1 Fig). In the network A, and B the I^2^ statistic was zero, and in the network C the I^2^ was 16.1% (S3 Table).

In network A, when compared to the Ultraflex stent, the polyflex stent increased the risk of stent migration 2.07 times (95% CI: 1.01, 4.67). The risk of stent migration for covered Evolution® stent, Flamingo stent, and Ultraflex stent plus radiotherapy was lower than the ultraflex stent; however, the 95% CIs involved the null values. In network B, the risk ratio for the latex prosthesis and plastic stents compared to the metallic stent was 6.82 (95% CI: 0.36, 127.54) and 2.87 (95% CI: 0.87, 10.64), respectively. In network C, there were no considerable differences between the conventional, Irradiation, open and ultraflex stents plus omeprazole and the Antireflux stent (S2 Fig). The results of simultaneous direct and indirect comparisons of treatments for stent migration are shown in S3 Table.

In terms of ranking, the covered Evolution® (p-score = 0.78), Uncovered (p-score = 0.88), and Irradiation stents (p-score = 0.65) were the best treatments in networks A, B and C, respectively (Table 2).

### Aspiration

Nine RCTs reported aspiration as a complication of stent placement. These RCTs involved 805 esophageal cancer patients. Three RCTs involved independent comparisons, so they were excluded from the network meta-analysis [20, 21, 27]. These studies have been described in Table 1. The remaining RCTs involved three independent networks (S3 Fig). The I^2^ statistic for all networks of this complication was zero (S4 Table). Forest plots drawn to compare the treatments with reference treatments in each network are shown in S4 Fig. Results of simultaneous direct and indirect comparisons of treatments for aspiration are shown in S2 Table. In terms of ranking, the Polyflex stent (p-score = 0.69), Irradiation stent (p-score = 0.74) and BT (p-score = 0.69) were the better treatments in networks A, B and C (Table 2).

### Severe pain

Severe pain was reported in 14 RCTs. The CSENACS (p-score = 0.73), Polyflex stent (p-score = 0.79), Latex prosthesis (p-score = 0.96) and BT (p-score = 0.65) were better treatments in terms of lower risk of severe pain among patients in networks A, B, C and D, respectively. The network and forest plots are shown in S5 and S6 Figs. According to the results of test for heterogeneity, The I^2^ statistic for network A, B, C, and D was zero (S5 Table). In addition, the results of direct and indirect comparisons are presented in S2 Table.

### Fistula formation

Fistula formation was reported in 10 RCTs. The networks of interventions and forest plots drawn to compare the treatments with reference treatments in each network are shown in S7 and S8 Figs, respectively. The I^2^ statistic for all networks of this complication was zero (S6 Table). The Plastic stent (p-score = 0.81), Conventional stent (p-score = 0.72), and SEMS 18 (p-score = 0.62) were better treatments in terms of lower risk of fistula formation in networks A, B, and C, respectively (Table 2).

## Discussion

In this network meta-analysis, we compared the complications of palliative treatments including stents in patients with esophageal cancer. The treatments were ranked based on their lower risk of complications including TRD, bleeding, stent migration, aspiration, severe pain and fistula formation.

Based on our results, the TRD complication involved four networks. In network A, the latex prosthesis stent increased the risk of TRD 3.89 times when compared to the metallic stent. Thermal ablative therapy decreased the risk of TRD. In network B, there was no considerable difference between the covered Evolution®, Flamingo, and Ultraflex stents compared with the Ultraflex stent. In network C, when compared to brachytherapy, SEMS 18 increased the risk of TRD. In network D, the open stent and Ultraflex plus omeprazole versus antireflux stent increased the risk of TRD among patients with esophageal cancer.

Upon ranking treatments in terms of lower risk of TRD, thermal ablative therapy, covered Evolution®, brachytherapy and antireflux were better treatment interventions in the TRD networks, respectively. However, in network C, there was no considerable difference in the p-score of brachytherapy (p-score = 0.72) and SEMS (p-score = 0.69).

In terms of lower risk of bleeding, Thermal ablative therapy, Conventional stent, Covered Evolution® stent, and Brachytherapy were ranked as best treatments in networks A, B, C, and D, respectively. The results of bleeding complication were similar to those of TRD. Based on the lower risk of stent migration, the covered Evolution® stent, uncovered stent and irradiation stent were ranked as best treatments in the networks of this complication.

The risk of aspiration was lower for the polyflex stent, Irradiation stent, and brachytherapy in the networks of this complication. However, there was no considerable difference between the first (brachytherapy) and second (SEMS 18) ranked treatments in network C. The risk of severe pain in the CSENACS, polyflex stent, latex prosthesis and brachytherapy was lower than in the other treatments in the networks.

In terms of fistula formation, the plastic stent, conventional stent and SEMS 18 were better treatments in the networks of this complication. However, it should be noted the plastic stents compared with other stents, increased the risk of TRD, bleeding, stent migration and aspiration. Therefore, we could not recommend plastic stents as a better treatment, just because the risk of fistula was lower for this stent. In addition, a recently published clinical guideline recommends against for placement of plastic stents for palliation of esophageal strictures [37].

Based on the results of a traditional meta-analysis, the risk of TRD in palliative locoregional modalities was lower than in the metallic stent (RR = 0.58, 95% CI: 0.17, 1.99). Moreover, the odds ratio (OR) for bleeding was 0.54 (95% CI: 0.29, 1.00) compared to the metallic stents [38]. These results are in line with ours.

In the aforementioned meta-analysis [38], the odds ratio of stent migration in patients who had received ultraflex stents versus other types of stents was 1.17 (95% CI: 0.71, 1.93). In our study, the risk of stent migration was higher for ultraflex stent versus covered Evolution® stent, Flamingo stent, and Ultraflex stent plus radiotherapy. However, the risk of stent migration was higher in the polyflex stent than in the ultraflex stent (RR = 2.17(1.01, 4.67). Moreover, in our study, the risk of stent migration for the flamingo stent was lower than in the polyflex and ultraflex stents, a finding that is consistent with the aforementioned traditional meta-analysis.

According to this meta-analysis [38], the odds ratio of severe pain for the ultraflex stent versus the other stents was 0.52; 95% CI (0.19, 1.45). This result is in line with our study regarding the comparison of ultraflex versus covered Evolution® stent and Flamingo stent. However, the risk of severe pain was higher than in the polyflex stent.

The SEMS has been introduced as a selective palliative treatment in patients with esophageal cancer. The TRD in patients in which this stent had been used was reported between 0 to 1.4% [4]. In our study, the SEMS and SEMS 18 were compared with brachytherapy in network C (for TRD) and in network D (for the bleeding complication). When compared with brachytherapy, SEMS 18 increased the risk of TRD and bleeding 5.61 and 2.62 times, respectively. Likewise, compared with brachytherapy, SEMS increased the risk of bleeding by 3 times. These findings confirm the available evidence regarding the role of brachytherapy in the palliative treatment of patients with advanced stages of esophageal cancer [39]. In addition, according to the results of a recently published meta-analysis brachytherapy was recommended as a relatively safe and highly effective treatment for palliation of dysphagia [40] that is in the line of our results in terms of lower risk of TRD and bleeding for this treatment. However, despite the available evidence regarding the safety of this treatment and strong recommendation of the international guidelines [37] for use of this treatment, the lack of experience of radiation oncologist lead to the underuse of brachytherapy [41].

According to the results of one RCT, the antireflux stent had no additional advantages over the open stent [42]. But in our study, the risk of TRD in patients who had received the open stent was more than in those who had received the antireflux stent. Moreover, the p-score for the antireflux and open stents were 0.74 and 0.36, respectively.

In our network meta-analysis, the confidence intervals for RRs involved the null value in some of the networks. The reason may be due to the low number of RCTs in each network. However, the point estimates of RR in the networks of complications were considerable.

The statistical heterogeneity among the networks of complications was low. However, the statistical power of the Cochrane test is low when the number of studies included is low [3]. So these results may have been affected by the low number of studies in the networks.

According to current literature, there is controversy over the selection of the ideal stent [4]. To our knowledge, our study is the first network meta-analysis to compare the available stents in terms of six major complications (treatment-related death, bleeding, stent migration, aspiration, severe pain and fistula formation) among patients with esophageal cancer. We have provided useful evidence on the ranking of stents with lower risks of the aforementioned complications. In addition, the advantage of our study -as a network meta-analysis- over the traditional meta-analysis is the simultaneous comparison of all available treatments in each network, while traditional meta-analysis compares the stents two by two or one stent versus another [38].

There are certain limitations in this study. Firstly, palliative treatments and stent interventions involved four networks of TRD, bleeding, and severe pain, and three networks for stent migration, aspiration, and fistula formation. Therefore, we could not compare all treatment interventions simultaneously for each complication in one network. Secondly, the number of included RCTs in each network was low, so the power of networks for estimating the indirect comparison was low and the confidence intervals for indirect estimates were wide [43], and this issue can be lead to spars-data bias [44]. Therefore, more RCTs are required for future network meta-analysis.

Secondly, because of the small number of RCTs in each network and consequently low power and lack of validity of statistical tests for publication bias [45, 46], we could not be assessed the publication bias in this network meta-analysis.

We recommend that future RCTs report ‘all’ the complications of stent placement and other palliative treatments in patients with esophageal cancer. Thus, future systematic reviews and network meta-analyses will be able to compare stents in terms of all the major complications simultaneously.

## Conclusion

Overall, the results of this network meta-analysis showed that thermal ablative therapy, covered Evolution® stents, brachytherapy and antireflux stents are associated with a lower risk of TRD. In terms of lower risk of bleeding, thermal ablative therapy, conventional stent, covered Evolution® stent and brachytherapy were better palliative treatments for patients with esophageal cancer. Based on the lower risk of stent migration, the covered Evolution®, uncovered, and Irradiation stents were better treatments. In terms of lower risk of severe pain as another major complication the CSENACS, polyflex stent, latex prosthesis and brachytherapy were better treatments.

## Supporting information

S1 File PRISMA checklist. (DOCX)

S2 File Search strategy for Medline. (DOCX)

S1 Fig Network of stent interventions for palliative treatments that reported the stent migration in esophageal cancer; A: Ultraflex stent is reference treatment, B: Metallic stent is reference, and C: Antireflux stent is reference.
(TIF)

S2 Fig Forest plots for stent migration in network A, B, C; Ultraflex stent, Metallic stent, Antireflux stent are reference treatments in network A, B, and C respectively.
(TIF)

S3 Fig Network of stent interventions for palliative treatments that reported the aspiration in patients; A: Ultraflex stent is reference treatment, B: Irradiation stent is reference, and C: Brachytherapy is reference.
(TIF)

S4 Fig Forest plots for aspiration in network A, B, C; covered evolution stent, Irradiation stent, brachytherapy are reference treatments in network A, B, and C respectively.
(TIF)

S5 Fig Network of stent interventions for palliative treatments that reported the severe pain in patients; Irradiation stent, Polyflex stent, Metallic stent and SEMS 18 are reference treatments in networks A, B, C and D respectively.
(TIF)

S6 Fig Forest plots for severe pain in network A, B, C and D; Antireflux stent, Ultraflex stent, Metallic stent, and brachytherapy are reference treatments in network A, B, C and D respectively.
(TIF)

S7 Fig Network of stent interventions for palliative treatments that reported the fistula in patients; Irradiation stent, Polyflex stent, Metallic stent and SEMS 18 are reference treatments in networks A, B, C and D respectively.
(TIF)

S8 Fig Forest plots for fistula in network A, B, and C. Metallic stent, Irradiation stent, and brachytherapy are reference treatments in network A, B, and C respectively.
(TIF)

S9 Fig Adjusted network funnel plot for network meta-analysis of TRD. (TIF)

S10 Fig Adjusted network funnel plot for network meta-analysis of bleeding complication. (TIF)

S11 Fig Adjusted network funnel plot for network meta-analysis of stent migration complication. (TIF)

S12 Fig Adjusted network funnel plot for network meta-analysis of aspiration complication. (TIF)

S13 Fig Adjusted network funnel plot for network meta-analysis of severe pain complication. (TIF)

S14 Fig Adjusted network funnel plot for network meta-analysis of fistula complication. (TIF)

S1 Table Simultaneous comparisons of palliative treatments using relative risk (95% CI) in terms of treatment related death among esophageal cancer patients. (DOCX)

S2 Table Simultaneous comparisons of palliative treatments using relative risk (95% CI) in terms of bleeding complications among esophageal cancer patients. (DOCX)

S3 Table Simultaneous comparisons of palliative treatments using relative risk (95% CI) in terms of stent migration among esophageal cancer patients. (DOCX)

S4 Table Simultaneous comparisons of palliative treatments using relative risk (95% CI) in terms of aspiration among esophageal cancer patients. (DOCX)

S5 Table Simultaneous comparisons of palliative treatments using relative risk (95% CI) in terms of severe pain among esophageal cancer patients. (DOCX)

S6 Table Simultaneous comparisons of palliative treatments using relative risk (95% CI) in terms of fistula among esophageal cancer patients. (DOCX)
