# Supplementary material for: Complications of stent placement in patients with esophageal cancer: A systematic review and network meta-analysis
Source: PLoS One. 2017 Oct 2;12(10):e0184784. doi: 10.1371/journal.pone.0184784 (PMC5624586; doi:10.1371/journal.pone.0184784)
Supplement: S2 File — (DOCX) [file pone.0184784.s002.docx]

Search strategy

#1: Esophageal cancer [tw]

#2: Esophageal squamous cell carcinoma [Mesh terms]

#3: Esophageal Neoplasms [Mesh terms]

#4: #1 OR #2 OR #3

#5: Randomized controlled trial [Mesh terms]

#6: Randomized clinical trial [tw]

#7: #5 OR #6

#8: Radiotherapy [Mesh terms]

#9: Stents [Mesh terms]

#10: Brachytherapy [Mesh terms]

#11: Palliative Care [Mesh terms]

#12: #8 OR #9 OR #10 OR #11

#13: #4 AND #7 AND #12
