# Supplementary figures and images for: Complications of stent placement in patients with esophageal cancer: A systematic review and network meta-analysis
Source: PLoS One. 2017 Oct 2;12(10):e0184784. doi: 10.1371/journal.pone.0184784 (PMC5624586; doi:10.1371/journal.pone.0184784)

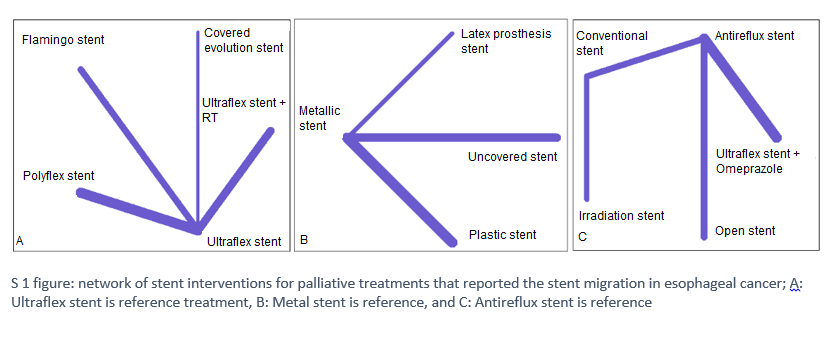

Supplement: S1 Fig — Network of stent interventions for palliative treatments that reported the stent migration in esophageal cancer; A: Ultraflex stent is reference treatment, B: Metallic stent is reference, and C: Antireflux stent is reference. (TIF) [file pone.0184784.s003.tif]

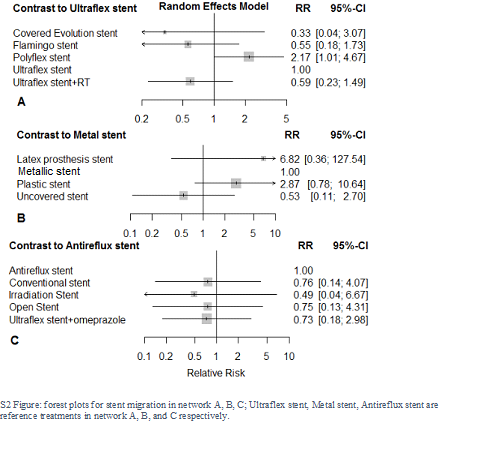

Supplement: S2 Fig — Forest plots for stent migration in network A, B, C; Ultraflex stent, Metallic stent, Antireflux stent are reference treatments in network A, B, and C respectively. (TIF) [file pone.0184784.s004.tif]

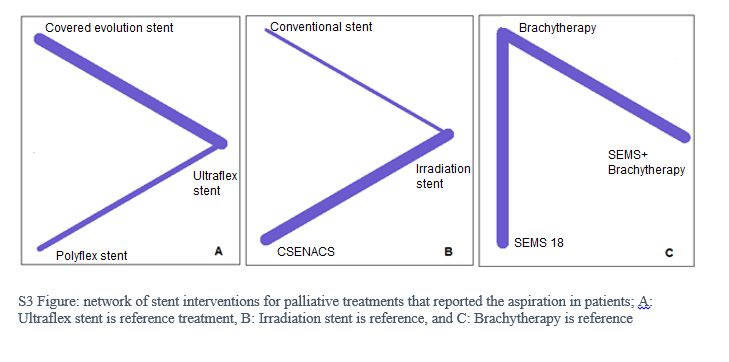

Supplement: S3 Fig — Network of stent interventions for palliative treatments that reported the aspiration in patients; A: Ultraflex stent is reference treatment, B: Irradiation stent is reference, and C: Brachytherapy is reference. (TIF) [file pone.0184784.s005.tif]

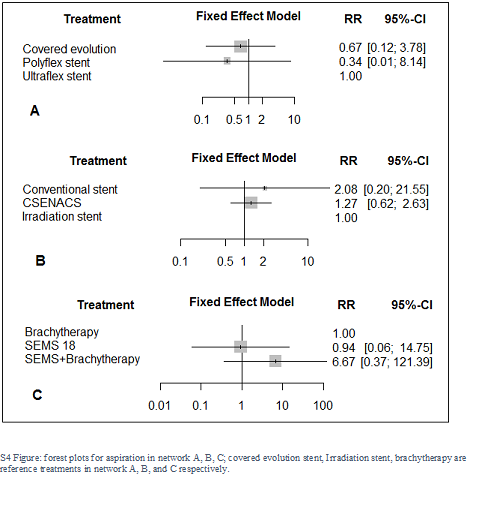

Supplement: S4 Fig — Forest plots for aspiration in network A, B, C; covered evolution stent, Irradiation stent, brachytherapy are reference treatments in network A, B, and C respectively. (TIF) [file pone.0184784.s006.tif]

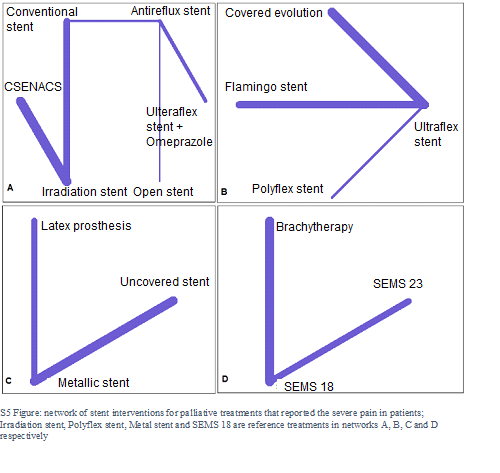

Supplement: S5 Fig — Network of stent interventions for palliative treatments that reported the severe pain in patients; Irradiation stent, Polyflex stent, Metallic stent and SEMS 18 are reference treatments in networks A, B, C and D respectively. (TIF) [file pone.0184784.s007.tif]

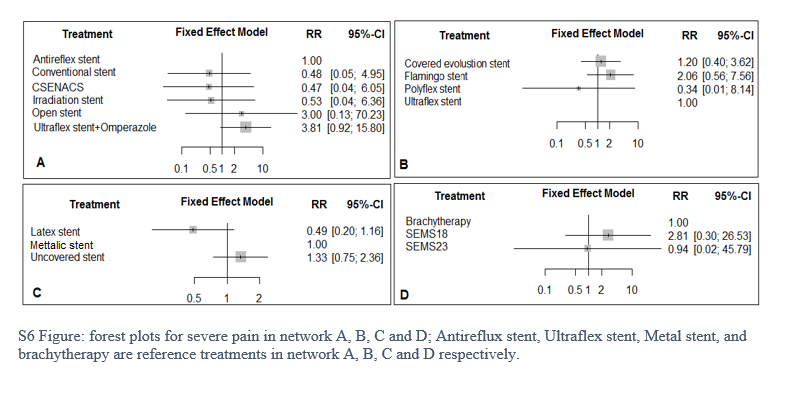

Supplement: S6 Fig — Forest plots for severe pain in network A, B, C and D; Antireflux stent, Ultraflex stent, Metallic stent, and brachytherapy are reference treatments in network A, B, C and D respectively. (TIF) [file pone.0184784.s008.tif]

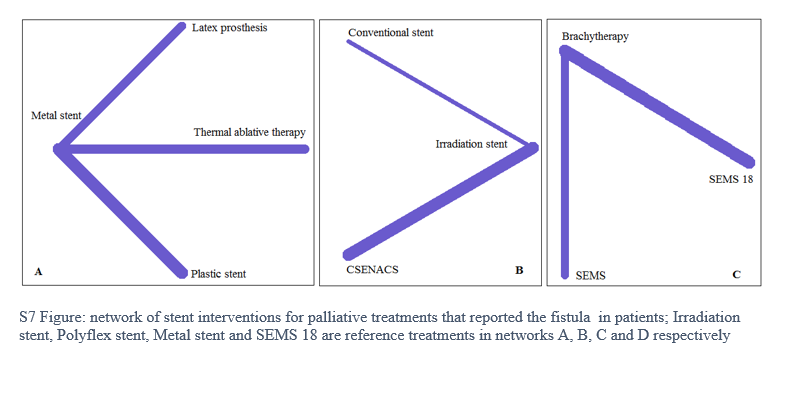

Supplement: S7 Fig — Network of stent interventions for palliative treatments that reported the fistula in patients; Irradiation stent, Polyflex stent, Metallic stent and SEMS 18 are reference treatments in networks A, B, C and D respectively. (TIF) [file pone.0184784.s009.tif]

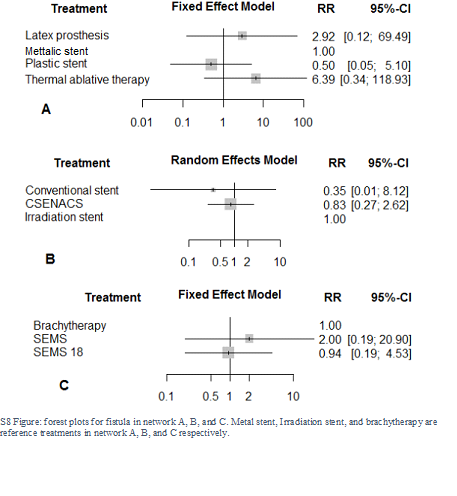

Supplement: S8 Fig — Forest plots for fistula in network A, B, and C. Metallic stent, Irradiation stent, and brachytherapy are reference treatments in network A, B, and C respectively. (TIF) [file pone.0184784.s010.tif]

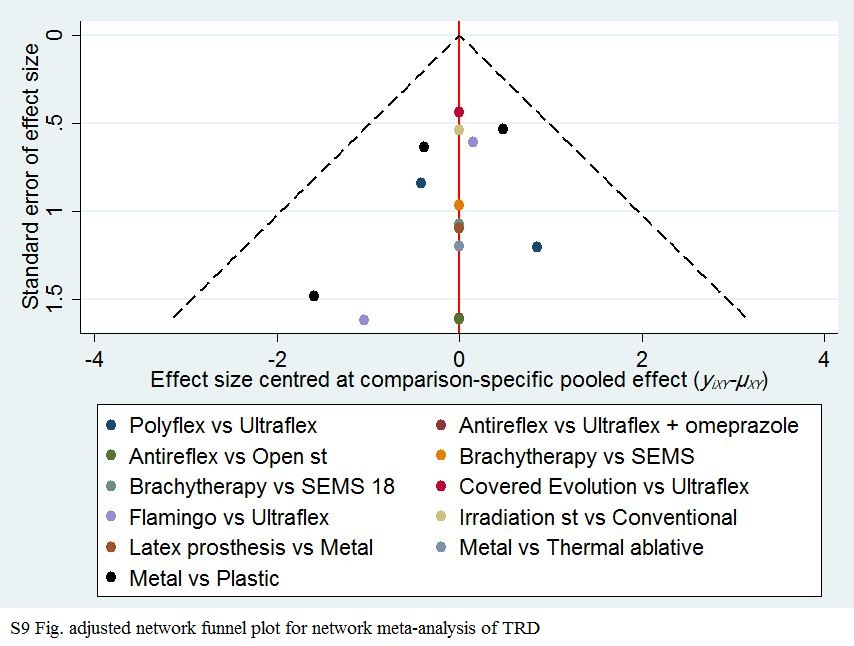

Supplement: S9 Fig — (TIF) [file pone.0184784.s011.tif]

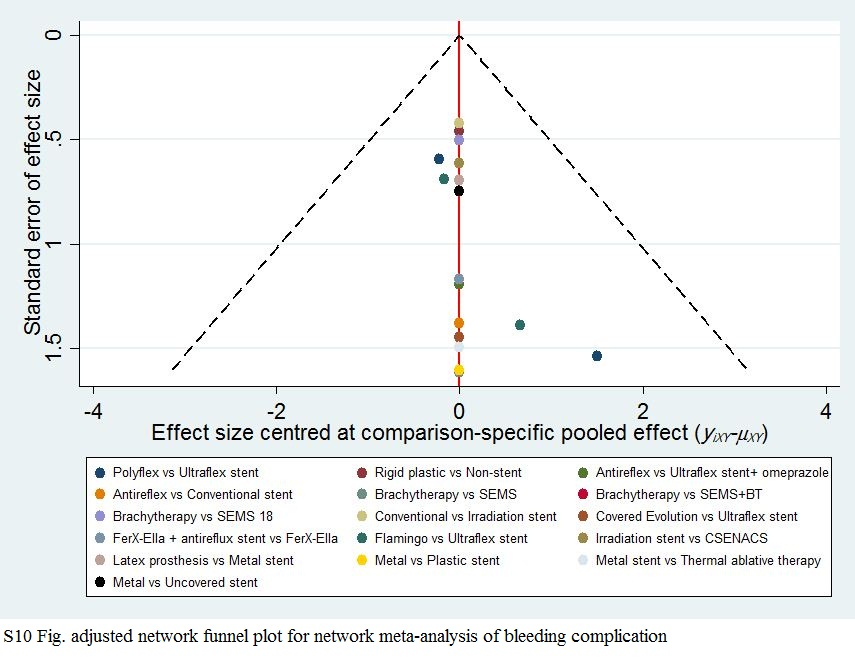

Supplement: S10 Fig — (TIF) [file pone.0184784.s012.tif]

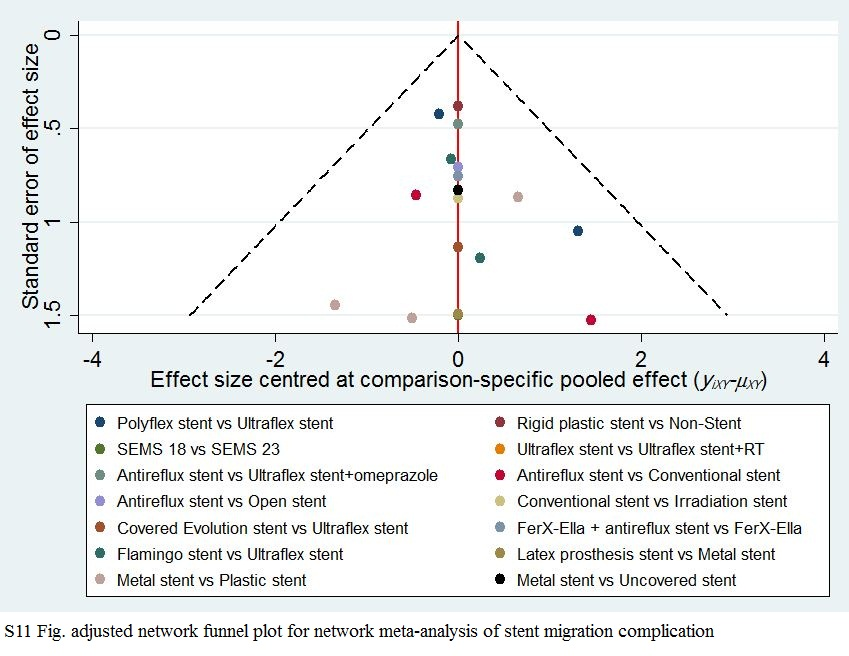

Supplement: S11 Fig — (TIF) [file pone.0184784.s013.tif]

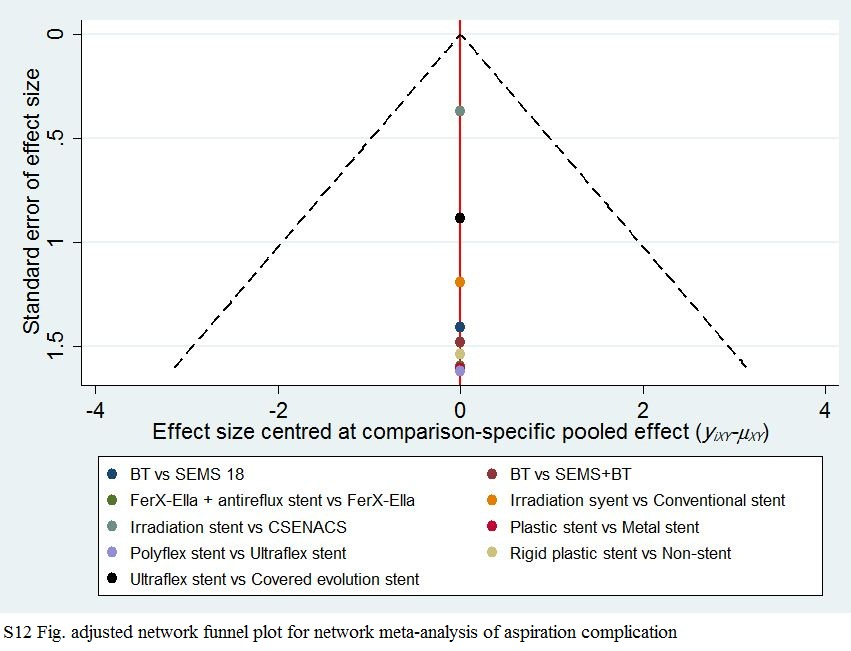

Supplement: S12 Fig — (TIF) [file pone.0184784.s014.tif]

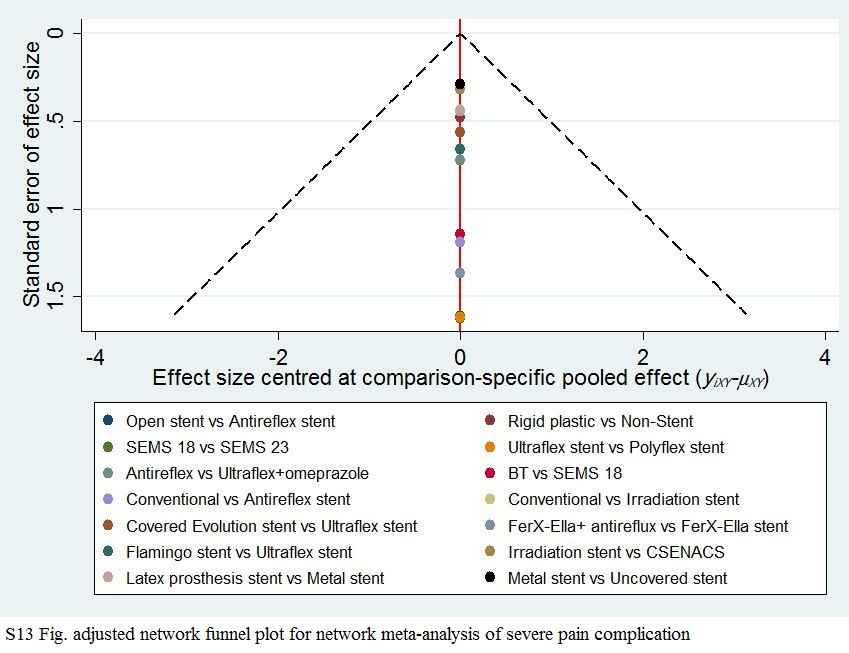

Supplement: S13 Fig — (TIF) [file pone.0184784.s015.tif]

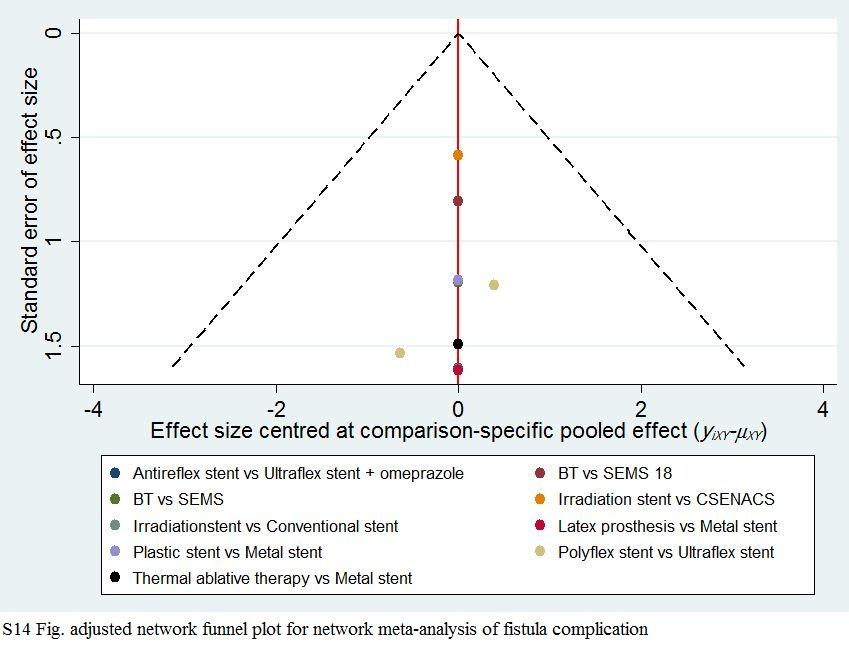

Supplement: S14 Fig — (TIF) [file pone.0184784.s016.tif]
