# Supplementary material for: Complications of stent placement in patients with esophageal cancer: A systematic review and network meta-analysis
Source: PLoS One. 2017 Oct 2;12(10):e0184784. doi: 10.1371/journal.pone.0184784 (PMC5624586; doi:10.1371/journal.pone.0184784)
Supplement: S1 Table — (DOCX) [file pone.0184784.s017.docx]

S1 Table: simultaneous comparisons of palliative treatments using relative risk (95% CI) in terms of treatment related death among esophageal cancer patients

| Network |  | Latex prosthesis | Metallic stent | Plastic stent | Thermal ablative therapy |
| --- | --- | --- | --- | --- | --- |
| A  tau2 = 0.1496;  I2 = 15.2%  Q=2.36  d.f=2  p-value=0.307 | Latex prosthesis |  | 4.24 (0.45, 39.82) | 2.77 (0.24, 31.71) | 9.64 (0.35, 267.01) |
|  | Metallic stent | 0.24(0.03, 2.22) |  | 0.65 (0.25, 1.71) | 2.28 (0.20, 26.42) |
|  | Plastic stent | 0.36 (0.03, 4.14) | 1.53 (0.58, 4.00) |  | 3.48 (0.25, 48.48) |
|  | Thermal ablative therapy | 0.10 (0.00, 2.87) | 0.44 (0.04, 5.10) | 0.29 (0.02, 4.00) |  |
| B  tau2 = 0;  I2 = 0%  Q=1.23  d.f=2  p-value=0.5409 |  | Covered evolution | Flamingo | Polyflex | Ultraflex |
|  | Covered evolution | . | 0.68 (0.12, 3.74) | 0.73 (0.12, 4.36) | 0.64 (0.22, 1.88) |
|  | Flamingo | 1.48 (0.27, 8.15) | . | 1.08 (0.16, 7.49) | 0.94 (0.25, 3.52) |
|  | Polyflex | 1.37 (0.23, 8.17) | 0.93 (0.13, 6.44) | . | 0.87 (0.21, 3.6) |
|  | Ultraflex | 1.57 (0.53, 4.65) | 1.06 (0.28, 3.98) | 1.15 (0.28, 4.74) | . |
| C  tau2 = 0;  I2 = 0%  Q =0  d.f=0  p-value= -- |  | Brachytherapy | SEMS | SEMS18 |  |
|  | Brachytherapy | . | 1.00 (0.13, 7.6) | 0.17 (0.02, 1.44) |  |
|  | SEMS | 1.00 (0.13, 7.6) | . | 0.17 (0.01, 3.23) |  |
|  | SEMS18 | 5.88 (0.7, 0.49.84) | 5.88 (0.31, 111.84) | . |  |
| D  Tau2=0  I2=0  Q=0  d.f=0  p-value= -- |  | Antireflux | Open stent | Ultraflex stent + omeprazole |  |
|  | Antireflux | . | 0.32 (0.01, 8.25) | 0.38 (0.01, 9.77) |  |
|  | Open stent | 3.12 (0.12, 80.39) | . | 1.18 (0.01, 116.80) |  |
|  | Ultraflex + omperazol | 2.65 (0.10, 68.30) | 0.85 (0.01, 83.94) | . |  |
