# Supplementary material for: Complications of stent placement in patients with esophageal cancer: A systematic review and network meta-analysis
Source: PLoS One. 2017 Oct 2;12(10):e0184784. doi: 10.1371/journal.pone.0184784 (PMC5624586; doi:10.1371/journal.pone.0184784)
Supplement: S2 Table — (DOCX) [file pone.0184784.s018.docx]

S2 table: simultaneous comparisons of palliative treatments using relative risk (95% CI) in terms of bleeding complications among esophageal cancer patients

| Network |  | Latex prosthesis | Metallic stent | Plastic stent | Thermal ablative therapy | Uncovered stent |
| --- | --- | --- | --- | --- | --- | --- |
| A  tau2 = 0  I2 = 0%  Q =0  d.f. =2  p-value=  -- | Latex | . | 1.62 (0.42 -6.31) | 0.57 (0.02 -17.4) | 12.44 (0.5 -312.48) | 6.09 (0.82 -44.93) |
|  | Metallic stent | 0.62 (0.16 -2.4) | . | 0.35 (0.02 -8.1) | 7.67 (0.41 -142.66) | 3.75 (0.86 -16.26) |
|  | Plastic stent | 1.76 (0.06 -53.81) | 2.85 (0.12 -65.93) | . | 21.88 (0.3 -1596.84) | 10.7 (0.33 -342.49) |
|  | Thermal ablative therapy | 0.08 (0 -2.02) | 0.13 (0.01 -2.43) | 0.05 (0 -3.34) | . | 0.49 (0.02 -12.88) |
|  | Uncovered stent | 0.16 (0.02 -1.21) | 0.27 (0.06 -1.16) | 0.09 (0 -2.99) | 2.04 (0.08 -53.85) | . |
| B  Tau2 = 0  I2 = 0%  Q= 0  d.f.= 0  p-value=  -- |  | Antireflux | Conventional stent | CSENACS* | Irradiation stent | Ultraflex stent+ Omeprazole |
|  | Antireflux | . | 1.16 (0.08 -17.28) | 0.94 (0.04 -20.17) | 0.94 (0.06 -15.8) | 0.59 (0.06 -6.09) |
|  | Conventional | 0.86 (0.06 -12.89) | . | 0.81 (0.19 -3.47) | 0.81 (0.35 -1.85) | 0.51 (0.01 -18.13) |
|  | CSENACS | 1.07 (0.05 -23.06) | 1.24 (0.29 -5.32) | . | 1 (0.3 -3.32) | 0.63 (0.01 -29.88) |
|  | Irradiation stent | 1.07 (0.06 -18.06) | 1.24 (0.54 -2.83) | 1 (0.3 -3.32) | . | 0.63 (0.02 -24.67) |
|  | Ultraflex stent + Omeprazole | 1.69 (0.16 -17.43) | 1.96 (0.06 -69.61) | 1.58 (0.03 -74.86) | 1.58 (0.04 -61.81) | . |
| C  tau2 = 0  I2 = 0  Q=1.38  d.f. =2  p-value=  0.5022 |  | Covered evolution | Flamingo | Polyflex | Ultraflex |  |
|  | Covered evolution | . | 0.09 (0 -1.99) | 0.05 (0 -1.08) | 0.07 (0 -1.13) |  |
|  | Flamingo | 10.91 (0.5 -236.63) | . | 0.57 (0.11 -2.88) | 0.73 (0.22 -2.44) |  |
|  | Polyflex | 19.21 (0.93 -397.7) | 1.76 (0.35 -8.93) | . | 1.28 (0.43 -3.79) |  |
|  | Ultraflex | 15 (0.89 -254.03) | 1.37 (0.41 -4.6) | 0.78 (0.26 -2.31) | . |  |
| D  Tau2 = 0  I2 = 0%  Q= 0  d.f.= 0  p-value=  -- |  | Brachytherapy | SEMS | SEMS18 | SEMSBT |  |
|  | Brachytherapy | . | 0.33 (0.01 -7.86) | 0.38 (0.14 -1.02) | 0.35 (0.02 -8.1) |  |
|  | SEMS | 3 (0.13 -70.78) | . | 1.15 (0.04 -31.4) | 1.05 (0.01 -90.49) |  |
|  | SEMS18 | 2.62 (0.98 -7.01) | 0.87 (0.03 -23.92) | . | 0.92 (0.03 -24.66) |  |
|  | SEMSBT | 2.86 (0.12 -66.28) | 0.95 (0.01 -82.26) | 1.09 (0.04 -29.42) | . |  |
| * CSENACS: Conventional self-expandable nitinol alloy covered stent | | | | | | |
