# Supplementary material for: Complications of stent placement in patients with esophageal cancer: A systematic review and network meta-analysis
Source: PLoS One. 2017 Oct 2;12(10):e0184784. doi: 10.1371/journal.pone.0184784 (PMC5624586; doi:10.1371/journal.pone.0184784)
Supplement: S3 Table — (DOCX) [file pone.0184784.s019.docx]

S3 Table: simultaneous comparisons of palliative treatments using relative risk (95% CI) in terms of stent migration among esophageal cancer patients

| Network |  | Covered Evolution stent | Flamingo stent | Polyflex stent | Ultraflex stent | Ultraflex stent + RT |
| --- | --- | --- | --- | --- | --- | --- |
| A  Tau2 = 0  I2 = 0%  Q= 1.53  d.f.= 2  p-value=  0.3945 | Covered Evolution stent | - | 0.6(0.05 -7.28) | 0.15(0.01 -1.61) | 0.33(0.04 -3.07) | 0.57(0.05 -6.31) |
|  | Flamingo stent | 1.66(0.14 -20.16) | - | 0.26(0.06 -1.01) | 0.55(0.18 -1.73) | 0.94(0.22 -4.11) |
|  | Polyflex stent | 6.51(0.62 -68.19) | 3.91(0.99 -15.41) | - | 2.17(1.01 -4.67) | 3.69(1.1 -12.37) |
|  | Ultraflex stent | 3(0.33 -27.63) | 1.8(0.58 -5.62) | 0.46(0.21 -0.99) | - | 1.7(0.67 -4.33) |
|  | Ultraflex stent +RT | 1.76(0.16 -19.59) | 1.06(0.24 -4.61) | 0.27(0.08 -0.91) | 0.59(0.23 -1.49) | - |
| B  Tau2 = 0  I2 = 0%  Q= 1.86  d.f.= 2  p-value=  0.4644 |  | Latex prosthesis stent | Metallic stent | Plastic stent | Uncovered stent |  |
|  | Latex prosthesis stent | - | 6.82(0.36 -127.54) | 2.37(0.1 -58.72) | 12.7841(0.45 -363.77) |  |
|  | Metallic stent | 0.15(0.01 -2.74) | - | 0.35(0.09 -1.29) | 1.88(0.37 -9.5) |  |
|  | Plastic stent | 0.42(0.02 -10.42) | 2.87(0.78 -10.64) | - | 5.39(0.67 -43.32) |  |
|  | Uncovered stent | 0.08(0 -2.23) | 0.53(0.11 -2.7) | 0.19(0.02 -1.49) | - |  |
| C  tau2 = 0.2932  I2 = 16.1%  Q=1.19  d.f. =1  p-value=  0.2749 |  | Antireflux stent | Conventional stent | Irradiation stent | Open stent | Ultraflex stent+ omeprazole |
|  | Antireflux stent | - | 1.32(0.25 -7.13) | 2.06(0.15 -28.36) | 1.33(0.23 -7.67) | 1.38(0.34 -5.66) |
|  | Conventional stent | 0.76(0.14 -4.07) | - | 1.56(0.21 -11.62) | 1.01(0.09 -11.42) | 1.04(0.12 -9.38) |
|  | Irradiation stent | 0.49(0.04 -6.67) | 0.64(0.09 -4.79) | - | 0.65(0.03 -15.12) | 0.67(0.03 -13.14) |
|  | Open stent | 0.75(0.13 -4.31) | 0.99(0.09 -11.25) | 1.55(0.07 -36.13) | - | 1.03(0.11 -9.79) |
|  | Ultraflex stent + omeprazole | 0.73(0.18 -2.98) | 0.96(0.11 -8.64) | 1.49(0.08 -29.36) | 0.97(0.1 -9.16) | - |
