# Supplementary material for: Complications of stent placement in patients with esophageal cancer: A systematic review and network meta-analysis
Source: PLoS One. 2017 Oct 2;12(10):e0184784. doi: 10.1371/journal.pone.0184784 (PMC5624586; doi:10.1371/journal.pone.0184784)
Supplement: S4 Table — (DOCX) [file pone.0184784.s020.docx]

S4 Table: simultaneous comparisons of palliative treatments using relative risk (95% CI) in terms of aspiration among esophageal cancer patients

| Network |  | Covered evolution | Polyflex stent | Ultraflex stent |
| --- | --- | --- | --- | --- |
| A  tau^2 = 0;  I^2 = 0%  Q=0, df=0 | Covered evolution | - | 1.95 (0.05 -72.57) | 0.67 (0.12 -3.78) |
|  | Polyflex stent | 0.51 (0.01 -19.03) | - | 0.34 (0.01 -8.14) |
|  | Ultraflex stent | 1.5 (0.26 -8.5) | 2.93 (0.12 -69.87) | - |
| B  tau^2 = 0;  I^2 = 0%  Q=0, d.f =0 |  | Conventional stent | CSENACS | Irradiation stent |
|  | Conventional stent | - | 1.63 (0.14 -18.9) | 2.08 (0.2 -21.55) |
|  | CSENACS | 0.61 (0.05 -7.1) | - | 1.27 (0.62 -2.63) |
|  | Irradiation stent | 0.48 (0.05 -4.99) | 0.79 (0.38 -1.62) | - |
| C:  tau^2 = 0;  I^2 = 0%  Q=0, d.f =0 |  | Brachytherapy | SEMS18 | SEMSBT |
|  | Brachytherapy | - | 1.07 (0.07 -16.87) | 0.15 (0.01 -2.73) |
|  | SEMS18 | 0.94 (0.06 -14.75) | - | 0.14 (0 -7.67) |
|  | SEMSBT | 6.67 (0.37 -121.39) | 7.14 (0.13 -390.82) | - |
