# Supplementary material for: Complications of stent placement in patients with esophageal cancer: A systematic review and network meta-analysis
Source: PLoS One. 2017 Oct 2;12(10):e0184784. doi: 10.1371/journal.pone.0184784 (PMC5624586; doi:10.1371/journal.pone.0184784)
Supplement: S5 Table — (DOCX) [file pone.0184784.s021.docx]

S5 Table: simultaneous comparisons of palliative treatments using relative risk (95% CI) in terms of severe pain among esophageal cancer patients

| Network |  | Antireflux stent | Conventional stent | CSENACS | Irradiation stent | Open stent | Ultraflex stent + Omeprazole |
| --- | --- | --- | --- | --- | --- | --- | --- |
| A  tau^2 = 0; I^2 = 0%  Q=0,  d.f =0 | Antireflux stent | - | 2.08 (0.2 -21.5) | 2.15 (0.17 -27.86) | 1.89 (0.16 -22.77) | 0.33 (0.01 -7.8) | 0.26 (0.06 -1.09) |
|  | Conventional stent | 0.48 (0.05 -4.95) | - | 1.03 (0.36 -2.97) | 0.91 (0.38 -2.15) | 0.16 (0 -8.09) | 0.13 (0.01 -1.94) |
|  | CSENACS | 0.47 (0.04 -6.05) | 0.97 (0.34 -2.8) | - | 0.88 (0.47 -1.64) | 0.16 (0 -9.04) | 0.12 (0.01 -2.3) |
|  | Irradiation stent | 0.53 (0.04 -6.36) | 1.1 (0.47 -2.6) | 1.13 (0.61 -2.11) | - | 0.18 (0 -9.77) | 0.14 (0.01 -2.44) |
|  | Open stent | 3 (0.13 -70.23) | 6.25 (0.12 -316) | 6.44 (0.11 -374.63) | 5.68 (0.1 -315.14) | - | 0.79 (0.02 -25.05) |
|  | Ultraflex + Omeprazole | 3.81 (0.92 -15.8) | 7.93 (0.52 -122.09) | 8.17 (0.44 -153.35) | 7.21 (0.41 -126.59) | 1.27 (0.04 -40.36) | - |
| B  tau^2 = 0; I^2 = 0%  Q=0,  d.f =0 |  | Covered evolution stent | Flamingo stent | Polyflex stent | Ultraflex stent |  |  |
|  | Covered evolution stent | - | 0.58 (0.11 -3.2) | 3.52 (0.12 -101.02) | 1.2 (0.4 -3.62) | - | - |
|  | Flamingo stent | 1.72 (0.31 -9.45) | - | 6.04 (0.2 -186.04) | 2.06 (0.56 -7.56) | - | - |
|  | Polyflex stent | 0.28 (0.01 -8.17) | 0.17 (0.01 -5.11) | - | 0.34 (0.01 -8.14) | - | - |
|  | Ultraflex stent | 0.83 (0.28 -2.51) | 0.49 (0.13 -1.78) | 2.93 (0.12 -69.87) | - | - | - |
| C  tau^2 = 0; I^2 = 0%  Q=0,  d.f =0 |  | Latex prosthesis | Metallic stent | Uncovered stent | - | - | - |
|  | Latex prosthesis | - | 0.49 (0.2 -1.16) | 0.37 (0.13 -1.03) | - | - | - |
|  | Metallic stent | 2.05 (0.86 -4.9) | - | 0.75 (0.42 -1.33) | - | - | - |
|  | Uncovered stent | 2.74 (0.97 -7.76) | 1.33 (0.75 -2.36) | - | - | - | - |
| D  tau^2 = 0; I^2 = 0%  Q=0, d.f =0 |  | Brachytherapy | SEMS18 | SEMS23 |  |  |  |
|  | Brachytherapy | - | 0.36 (0.04 -3.37) | 1.07 (0.02 -52.35) | - | - | - |
|  | SEMS18 | 2.81 (0.3 -26.53) | - | 3 (0.13 -71.91) | - | - | - |
|  | SEMS23 | 0.94 (0.02 -45.79) | 0.33 (0.01 -7.99) | - | - | - | - |
