# Supplementary material for: Complications of stent placement in patients with esophageal cancer: A systematic review and network meta-analysis
Source: PLoS One. 2017 Oct 2;12(10):e0184784. doi: 10.1371/journal.pone.0184784 (PMC5624586; doi:10.1371/journal.pone.0184784)
Supplement: S6 Table — (DOCX) [file pone.0184784.s022.docx]

S6 Table: simultaneous comparisons of palliative treatments using relative risk (95% CI) in terms of fistula among esophageal cancer patients

| Network |  | Latex prosthesis | Metallic stent | Plastic stent | Thermal ablative therapy |
| --- | --- | --- | --- | --- | --- |
| Net A:  tau^2 = 0; I^2 = 0%  Q=0,  d.f =0 | Latex prosthesis | . | 2.92 (0.12 -69.49) | 5.84 (0.11 -297.25) | 0.46 (0.01 -34.08) |
|  | Metallic stent | 0.34 (0.01 -8.14) | . | 2 (0.2 -20.41) | 0.16 (0.01 -2.91) |
|  | Plastic stent | 0.17 (0 -8.7) | 0.5 (0.05 -5.1) | . | 0.08 (0 -3.27) |
|  | Thermal ablative therapy | 2.19 (0.03 -163.06) | 6.39 (0.34 -118.93) | 12.78 (0.31 -534.96) | . |
| Net B:  tau^2 = 0; I^2 = 0%  Q=0,  d.f =0 |  | Conventional stent | CSENACS | Irradiation stent | - |
|  | Conventional stent | . | 0.42 (0.01 -11.92) | 0.35 (0.01 -8.12) | - |
|  | CSENACS | 2.41 (0.08 -69.17) | . | 0.83 (0.27 -2.62) | - |
|  | Irradiation stent | 2.89 (0.12 -67.86) | 1.2 (0.38 -3.77) | . | - |
| Net C:  tau^2 = 0; I^2 = 0%  Q=0,  d.f =0 |  | Brachytherapy | SEMS | SEMS18 | - |
|  | Brachytherapy | . | 0.5 (0.05 -5.22) | 1.07 (0.22 -5.18) | - |
|  | SEMS | 2 (0.19 -20.9) | . | 2.14 (0.13 -36.14) | - |
|  | SEMS18 | 0.94 (0.19 -4.53) | 0.47 (0.03 -7.9) | . | - |
